# A Readout Circuit for MEMS Gas Sensor

**DOI:** 10.3390/mi14010150

**Published:** 2023-01-06

**Authors:** Shengle Ren, Mingyuan Ren, Honghai Xu

**Affiliations:** 1 College of Electrical and Information Engineering, Quzhou Universiy, Quzhou 324000, China; 2 Jinhua Advanced Research Institute, Jinhua 321013, China; 3 School of Computer Science and Technology, Harbin University of Science and Technology, Harbin 150080, China

**Keywords:** MEMS gas sensor, interface circuit, ADC

## Abstract

In recent years, the application of gas sensors is becoming more and more extensive. Driven by potential applications such as the Internet of Things, its technology development direction begins with miniaturization, integration, modularization, and intelligence. However, there is a bottleneck in the research of interface circuits, which restricts the development of gas sensors in volume, power consumption, and intelligence. To solve this problem, a MEMS gas sensor interface circuit based on ADC technology is proposed in this paper. Under the condition of the Huahong 110 nm process, the working voltage is 3.3 V, the resistance change of 100 Ω~1 MΩ can be detected, the conversion error is in the range of 0.5~1%, and the maximum power consumption is 986 μW. The overall layout area is 0.49 × 0.77 mm^2^. Finally, the correctness of the circuit function is verified by post-layout simulation.

## 1. Introduction

The interface circuit is a key component as the conversion bridge between the gas sensor signal and the digital output signal. To a certain extent, its performance determines the various indicators of the gas sensor. The traditional interface circuit based on Analog digital conversion (ADC) technology has a complex design, large area, high power consumption, and high cost. However, the emergence of Micro-Electro-Mechanical System (MEMS) gas sensors means that the interface circuit can integrate “on-chip” ADCs, which is currently a research hotspot in the field of gas sensors.

In order to design low noise, low power consumption, small size and high performance (sensitivity), and multi-function MEMS gas sensor, its key module is still on the interface circuit research. Interface circuits can be divided into two basic categories [1], one based on pulse-width modulation (PWM) technology [2,3,4,5] and the other based on ADC technology [6,7,8,9,10].

Based on the basic principle of the interface circuit of PWM technology is as the sensor is sensitive to the resistance of the resistance changes, charge, or discharge of the capacitor, so as to get a different pulse width signal, finally through the comparator circuit that can be directly converted into digital signals, such as this kind of interface circuit without the ADC circuit, thus has simple structure, small chip area, low power consumption. Nevertheless, the basic principle of the interface circuit based on ADC technology is that when the sensor resistance changes, the input signal is amplified and filtered by the front-end circuit, and then an ADC circuit is cascaded to obtain digital output through analog-to-digital conversion. This kind of interface circuit has higher precision, better signal-to-noise ratio, and better adaptability in a special environment.

Sigma-delta ADCs and Successive Approximation Register (SAR) ADCs are widely used in sensor systems. The Sigma-delta ADC can achieve high precision with a lower bit quantizer due to its noise shaping capability, but it consumes a lot of power due to the existence of fixed direct current (DC) bias within op-amp based integrator [11]. By contrast, owing to the advantages of their simple structure and minimal usage of an analog circuit, SAR ADCs are well compatible with the increasingly scaled-down technology and operate at an ultra-low power supply [12].

In 2012, Ha proposed a CDS technology to reduce interface circuit noise and 12-bit SAR ADC with time-interleaved single differential sampling to reduce power consumption [13]. In 2016, Chiang used fourth-order continuous time (CT) Σ-Δ. The modulation (SDM) circuit quantizes and reduces the noise of the voltage signal, and finally obtains the digital output through a latch comparator [14]. In 2019, Dudina designed low-noise, wide-bandwidth, and wide-dynamic-range sensitive circuits by cascading two Programmable Gain Amplifier (PGA) circuits with different functions [15].

Most of the traditional research on interface circuits based on the ADC method uses “off-chip” ADC. Although it can also reduce power consumption and area, the dynamic range is often not high enough, which will affect the accuracy, and the power consumption that can reach a high dynamic range is often high. On the other hand, the transmission between the “off-chip” ADC and the front circuit will lose more power, and the “off-chip” structure also occupies a larger chip area. However, combined with MEMS technology, ADC can be integrated on the same chip as the front circuit, which can greatly reduce the chip area. Improved power utilization and ADC can directly collect data from input sources without the need for an additional controller to reconfigure or process it. Therefore, this paper proposes a MEMS gas sensor interface circuit based on SAR ADC with low power consumption, low noise, small size, and high precision.

## 2. Interface Circuit Architecture

**Figure 1:**
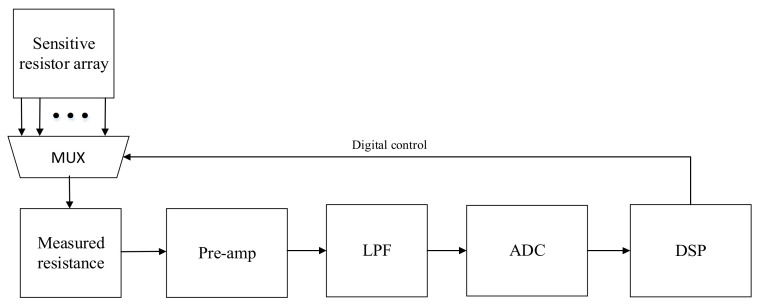
Schematic diagram of the basic structure of the interface circuit.

The common architecture of a MEMS gas sensor interface circuit is shown in Figure 1. The interface circuit is composed of sensor sensitive resistor array, multiplexer (MUX), pre-stage operational amplifier, low-pass filter (LPF) circuit, ADC circuit, and a digital signal processing circuit. Sensor sensitive resistor array: the module can select the appropriate sensitive resistor according to the actual needs (gas type, concentration). Front stage operational amplifier: the module reflects the change of resistance value by detecting the change of voltage or conduction current at both ends of the sensitive resistor, so as to extract the gas information (type and concentration) in the MEMS gas sensor, sort and adjust each input signal, and also act as a buffer stage to isolate the sensor and the back-end circuit. LPF: the module can filter signals of different frequencies to make useful frequency signals pass through, and suppress useless frequency signals, which can play a role in signal processing, data transmission, filtering interference noise, etc. ADC circuit: the module converts analog signals processed by the previous circuit into digital signals. Digital signal processing (DSP) circuit: the module controls the resistance matching of the multiplexer, ADC calibration, gain selection, or offset compensation of the pre-stage operational amplifier and other functions through the logic circuit to generate different time sequences.

### 2.1. Pre-Stage Circuit Design

**Figure 2:**
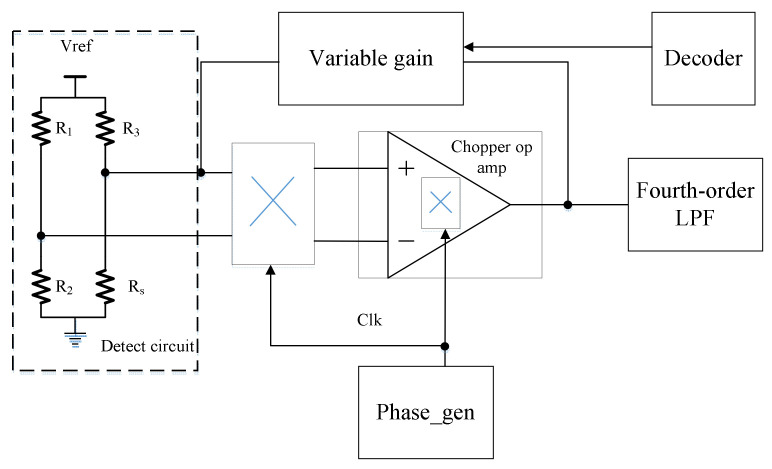
The basic structure of the previous circuit.

In order to meet the demand for gas sensors, chopper op adopts variable gain technology, and a high-order active low-pass filter circuit is cascaded at the output end to ensure low noise and certain linearity and gain accuracy, which is suitable for the detection of multiple sensitive sources. The overall architecture is shown in Figure 2. In the overall architecture of the front stage circuit, the Wheatstone Bridge structure is used as the detection circuit. R_1_, R_2,_ and R_3_ are reference resistors with fixed resistance values, and R_s_ are variable resistors, which are used to simulate changes in sensitive sources. Two kinds of chopper structures are used to chopper the input signal outside the amplifier, and the two structures with high noise contribution are in the internal circuit three times. Phase_Gen module is responsible for providing a control clock signal to the chopper circuit. The output signal generated by the Decoder module is used to control the Variable gain module to select the appropriate circuit gain according to the actual working needs of the circuit. The Sallen-key fourth-order active LPF circuit is cascaded to filter the high-frequency noise of the previous circuit.

**Figure 3:**
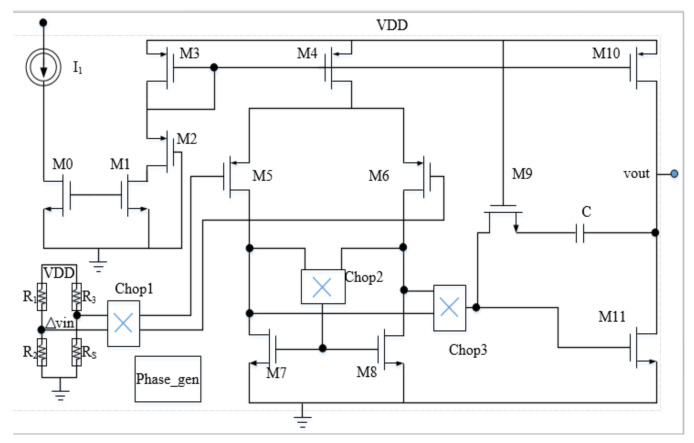
Schematic of low noise chopper operational amplifier circuit.

The low noise chopping operation amplifier circuit is shown in Figure 3. It is verified by simulation that the circuit noise is only 8 nV/sqrt (Hz), and the power consumption is 16.73 μW. The circuit has four modes of gain adjustment of 20 dB, 40 dB, 60 dB, and 80 dB. By cascading a fourth-order active low-pass filter circuit, a smooth curve with a unilateral output swing of 646.84 mV~2.64 V and an amplitude of 1.99 V can be obtained. The entire pre-stage circuit exhibits good gain linearity and noise reduction characteristics.

### 2.2. SAR ADC Circuit Design

**Figure 4:**
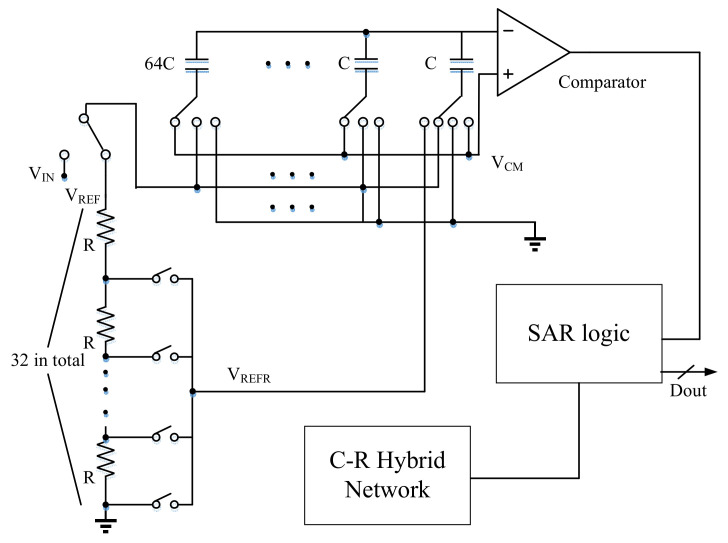
Topology diagram of 12-bit SAR ADC based on C-R network.

As shown in Figure 4, the 12-bit SAR ADC adopts a hybrid capacitance-resistor (C-R) DAC structure. It is composed of a 5-bit resistor voltage divider network and a 7-bit capacitor voltage divider network. Then compare the DAC output with the common mode voltage (Vcm) through a comparator within a certain time. The obtained results can be used to control the whole C-R switching network through the SAR logic module.

**Figure 5:**
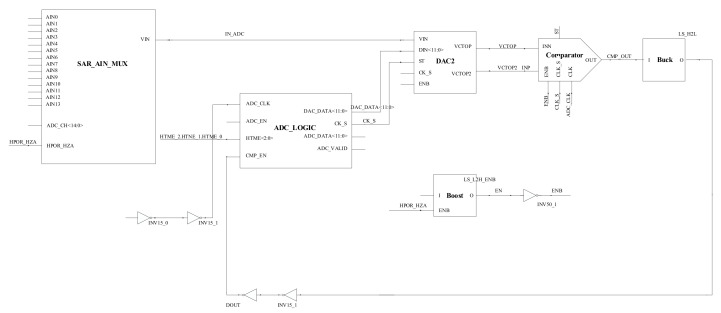
Circuit diagram of 12-bit SAR ADC.

As shown in Figure 5, the circuit schematic diagram of the 12-bit SAR ADC designed in this paper includes a MUX, a boost conversion circuit (Boost), and a buck conversion circuit (Buck) in addition to the comparator, DAC, and SAR Logic described previously. The multiplexer is used to simulate the sensing signal processed by the previous circuit and provide different input signals for ADC. A buck circuit can realize the conversion function of a voltage signal from 3 V to 1.5 V. Similarly, a boost circuit can realize the conversion function of a voltage signal from 1.5 V to 3 V. This paper aims to design a SAR ADC with low power consumption and small size, so the digital circuit accounts for a large proportion of SAR ADC. In addition, since the SAR ADC does not need to provide a large voltage signal during operation, unlike the analog circuit, boost and buck are designed to reduce the power consumption of the circuit in processing the analog signal and digital signal.

This paper aims to design a SAR ADC with low power consumption and a small size. Therefore, digital circuits account for a large proportion. Because they do not need to provide large voltage signals as analog circuits do when working, boost conversion circuits and buck conversion circuits are designed to reduce the power consumption of circuit processing analog signals and digital signals.

## 3. Results

**Figure 6:**
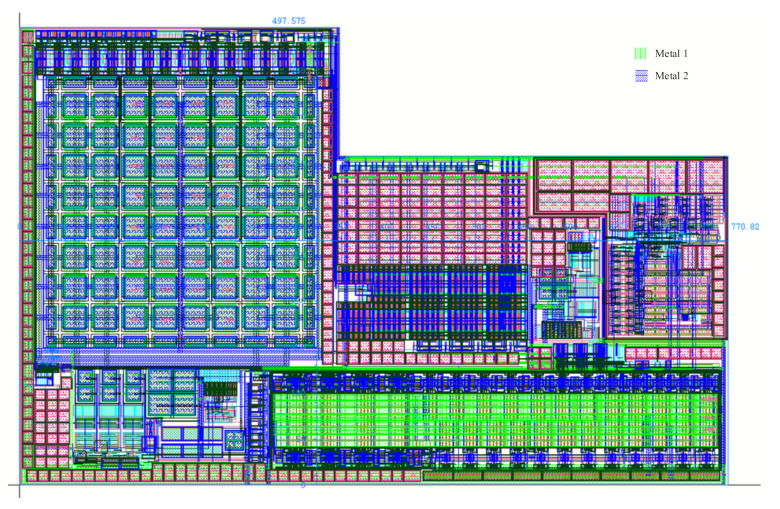
The overall layout design of the system.

Figure 6 shows the overall layout of the interface circuit system designed in this paper. The layout area is 497.57 × 770.82 μm^2^.

### 3.1. Interface Circuit Module Simulation and Analysis

**Figure 7:**
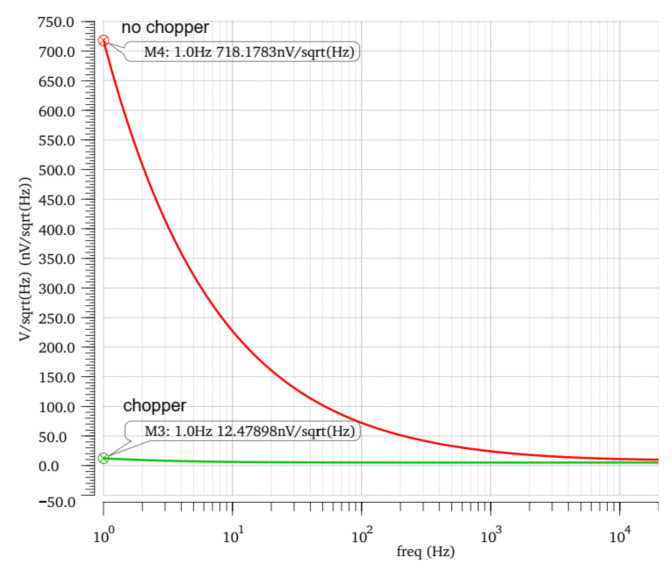
Noise simulation of chopper operational amplifier.

The simulation results after the layout of the front-stage operational amplifier are shown in Figure 7. At the low frequency of 1 Hz, the noise of the circuit with a chopper is 12.47 nV/sqrt (Hz), while the noise of the circuit without a chopper is 718.17 nV/sqrt (Hz), which still has good noise reduction capability. The simulation results of the front-stage operational amplifier are shown in Table 1.

**Figure 8:**
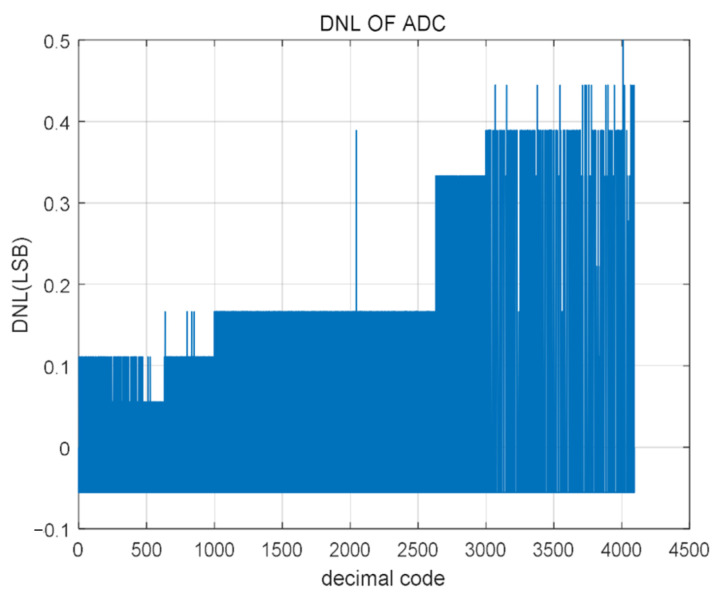
Simulation test results of DNL.

**Figure 9:**
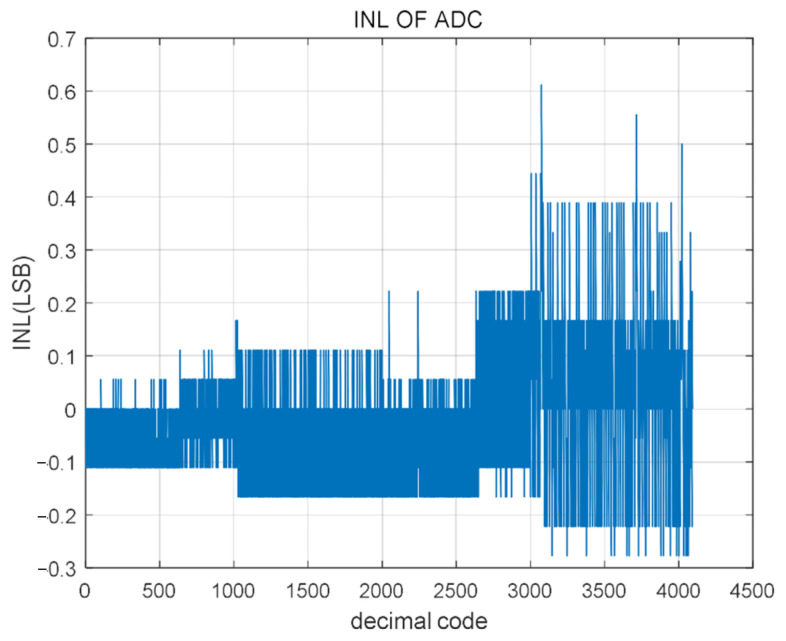
Simulation test results of INL.

The output signal FFT of the SAR ADC changes, as shown in Figure 8 and Figure 9. The corresponding Differential Non-linearity (DNL) and Integral Non-linearity (INL) post-simulation test results are, respectively. It can be seen from the figure that the range of DNL is −0.05 LSB/+0.5 LSB; The INL range is −0.28 LSB/+0.61 LSB. Although the error has increased, the change is not significant.

**Figure 10:**
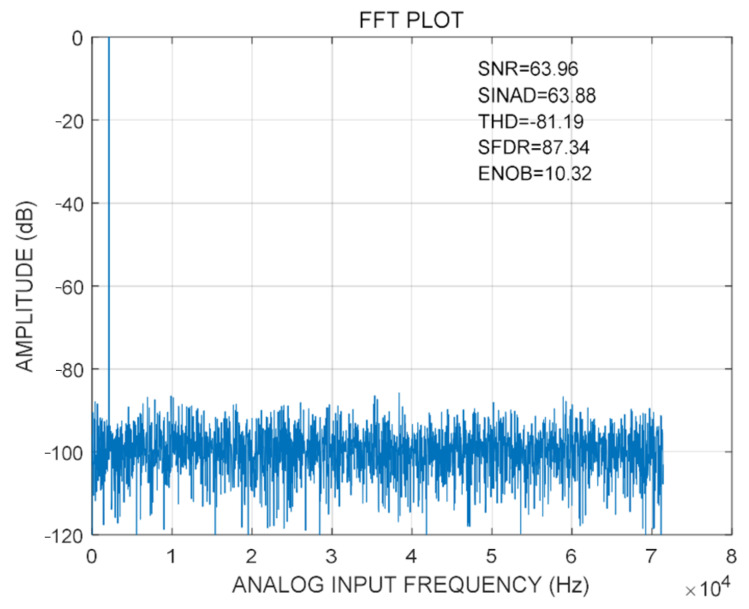
Simulation of 12-bit SAR ADC.

In order to verify the dynamic performance of the 12-bit SAR ADC after the layout, when the ADC sampling rate is 1 MSps, the frequency of the input signal is 2 kHz. A total of 4096 points are selected from the output signal for FFT analysis after the layout. The results are shown in Figure 10. The SNR of the designed 12-bit SAR ADC is 63.96 dB, SINAD is 63.88 dB, THD is 81.19 dB, SFDR is 87.34 dB, and ENOB is 10.32 bits, indicating that the dynamic performance of the designed ADC still meets the design requirements after passing the layout simulation.

### 3.2. System Simulation and Analysis

**Figure 11:**
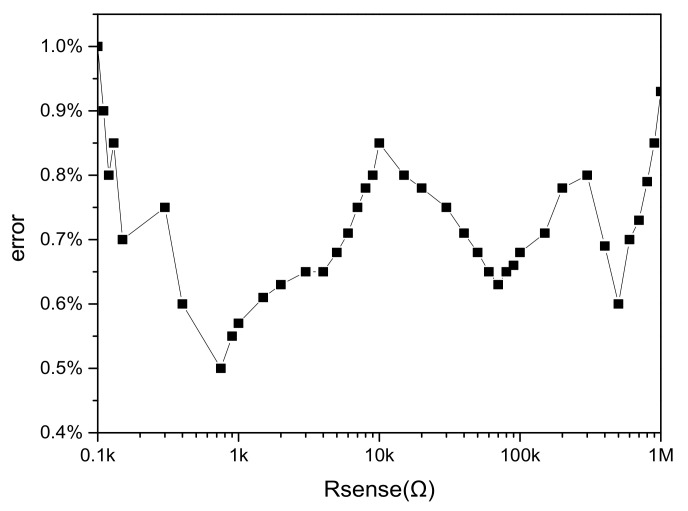
Simulation after system conversion error.

**Figure 12:**
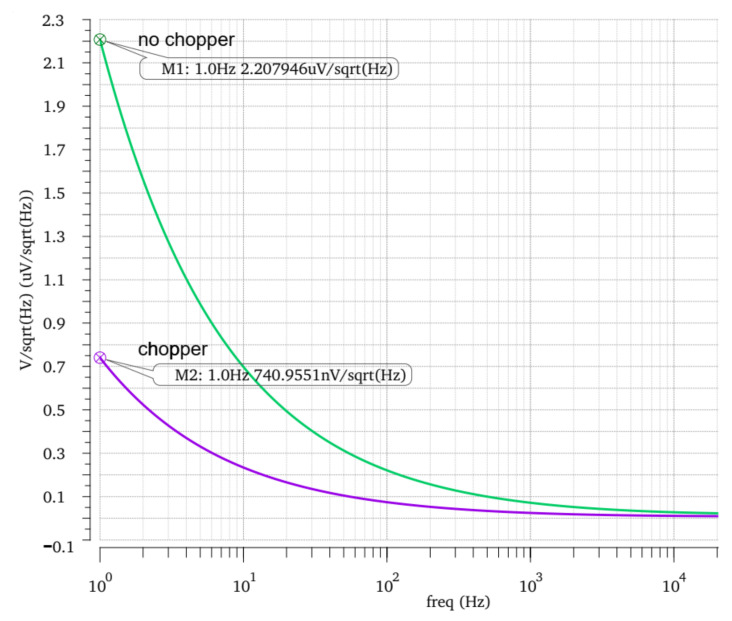
Post-simulation of system noise layout.

Then the system noise and conversion error are verified by post-simulation, and the simulation results are shown in Figure 11 and Figure 12, respectively. It can be found in Figure 11 that when the sensitive resistance is 100 Ω to 1 KΩ, the conversion error fluctuates up and down at some points, but the general trend still decreases with the increase of resistance value until the minimum error is 0.5%. When the sensitive resistance is from 1 KΩ to 10 KΩ, the conversion error increases with the increase of resistance value, and the maximum error is about 0.84%. When the sensitive resistance is between 10 KΩ and 100 KΩ, the conversion error decreases with the increase of resistance value until 0.63%. When the sensitive resistance is between 100 KΩ and 1 MΩ, the conversion error varies greatly, but is less than 1%. It can be seen that the measurement accuracy of the designed interface circuit system is the same as that of the previous simulation, both within the range of 0.5~1%. In Figure 12, the noise of the system using chopper technology is 740.95 nV/sqrt (Hz) at 1 Hz. The system noise without chopper technology is 2.20 μV/sqrt (Hz) at 1 Hz. The post-copy of the layout verifies the noise reduction capability of the front circuit to the interface circuit.

The research results of this paper are compared with other relevant studies at home and abroad, and the comparative results are shown in Table 2. It can be seen that the MEMS gas sensor interface circuit designed in this paper can actually measure the resistance changes of four orders of magnitude, and the measurement error is within the range of 0.5% to 1%. When the power supply is 3.3 V, the power consumption is only 0.98 mW, and the chip area is only 0.377 mm^2^. Compared with other studies, it has certain advantages in chip area and power consumption. The power consumption of the circuit is reduced by using Boost and Buck. The SAR logic array using a C-R hybrid structure is used to reduce the system area.

## 4. Conclusions

Based on Hua Hong’s 110 nm process, this paper proposes a MEMS gas sensor interface circuit based on ADC technology. The gas sensor interface circuit can be reduced by 1.16% without chopper technology and chopper technology μV/sqrt (Hz) system noise. The system can actually measure the resistance change from 100 Ω to 1 MΩ, and the conversion error is within the range of 0.5% to 1%. When the power supply is 3.3 V, the power consumption is only 0.98 mW. The layout area of the whole interface circuit is about 0.377 mm^2^. Through the comparison and analysis with related research, the interface circuit designed in this paper has certain advantages in chip area and power consumption.

## Figures and Tables

**Table 1:** Simulation results of pre stage operational amplifier.

Parametera	Performance
Open loop gain (dB)	62.76
Phase margin (°)	66
Cut off frequency (Hz)	338.93 K
Output voltage swing (V)	0.49~2.84
Input voltage range (V)	0~2.44
Swing rate (V/μs)	0.95
CMRR(dB)	75.5
PSRR(dB)	70.2
Power consumption (μW)	16.73

**Table 2:** Performance comparison of interface circuits.

	[8]	[16]	This Work
Technology (nm)	180	180	110
Voltage (V)	1.8	1.8	3.3
Sensing range (Ω)	80–2 M	1–500 M	100–1 M
Relative error (%)	<1.5	<1	0.5–1
Power consumption (mW)	1.8	7.92	0.98
Area (mm^2^)	0.64	0.996	0.377

## Data Availability

Not applicable.
